# *Amomum villosum* Lour. Polyphenols extract: Effective inhibitors of heterocyclic aromatic amines in grilled beef patties

**DOI:** 10.1016/j.fochx.2026.103947

**Published:** 2026-05-05

**Authors:** Yuhao Huang, Yanan Liu, Bao-Zhu Jia, Zhen-Lin Xu, Lin Luo

**Affiliations:** a Guangdong Provincial Key Laboratory of Food Quality and Safety, South China Agricultural University, Guangzhou 510642, China; b College of Biology and Food Engineering, Guangdong University of Education, Guangzhou 510303, China; c Guangdong Research Center for Rural Policy, South China Agricultural University, Guangzhou 510642, China

**Keywords:** Heterocyclic aromatic amines, *Amomum villosum* Lour., Inhibition mechanism, Phenolic compounds, Grilled beef patties

## Abstract

Polyphenols can inhibit heterocyclic aromatic amines (HAAs) formation. However, the inhibitory efficacy and mechanisms of polyphenols from different sources vary significantly against different types of HAAs. This study investigated the inhibitory effect of *Amomum villosum* Lour. polyphenols extract on the formation of HAAs and explored the underlying mechanisms. The highest inhibition rate of total HAAs (43.11%) was observed at an extract concentration of 0.5 mg/g, with significant reductions in nine key HAAs. *Amomum villosum* Lour. polyphenols extract can reduce the content of HAA precursors (amino acids, glucose, creatine) and decrease fat oxidation. The suppression of HAAs is attributed to two concerted mechanisms: the potent antioxidant capacity and the adduction of phenolic compound with reactive HAA intermediates, thereby quenching key reaction pathways. These findings provide a mechanistic basis for the application of *Amomum villosum* Lour. as a natural HAA inhibitor in roasted meat products.

## Highlights

• Effects of *A. villosum* polyphenols on formation of HAAs were first investigated.
• Suppresses HAAs by 43.11% while no impact on quality.
• *A. villosum* polyphenols can scavenges free radicals and binds HAAs intermediates.

## Graphical abstract

**Figure:**
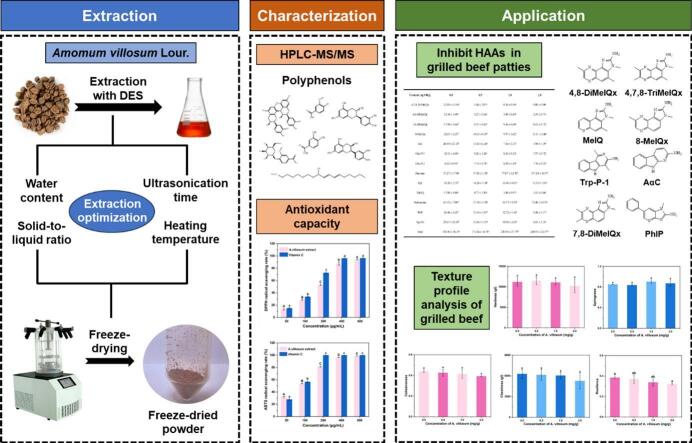

## 1 Introduction

Grilled meat is prepared through cutting, marinating, and cooking over an open flame, contact grill, or in an oven, which gives it a distinctive color and flavor. Although high-temperature grilling enhances taste, it can also generate harmful compounds, including HAAs. Since their first detection in 1977 using the Ames Salmonella mutagenicity assay (Gibis, 2016), HAAs have been widely recognized as carcinogenic and mutagenic agents (Kim & Hur, 2018). Studies also indicate that HAAs cause genotoxicity (Fuccelli et al., 2018), neurotoxicity (Lawana et al., 2020), and cardiotoxicity (Davis & Snyderwine, 1995). As a result, HAAs in grilled meat has raised significant safety concerns.

Common HAAs, such as imidazo[4,5-*f*]quinoxaline (IQ), 2-amino-3,4-dimethylimidazo[4,5-*f*]quinoline (MeIQ), 2-amino-1-methyl-6-phenylimidazo[4,5-*b*]pyridine (PhIP), 2-amino-9H-pyrido[2,3-*b*]indole (AαC), and 3-amino-1,4-dimethyl-5H-pyrido[4,3-*b*]indole (Trp-P-1), are generated through diverse and complex pathways. For example, IQ- and IQx-type HAAs arise mainly from Strecker degradation or Maillard reaction products of reducing sugars, which subsequently react with amino acids and creatinine (Wang et al., 2023). In contrast, PhIP forms from phenylalanine via phenylacetaldehyde, followed by aldol condensation with creatinine (Ding et al., 2025). Radicals such as formamide and alkyl radicals then promote cyclization into a pyridine structure (Meurillon & Engel, 2016). The formation of HAAs in grilled meat depends on several factors, including cooking temperature, time, ingredient type, and composition. Current mitigation approaches fall into three categories (Dong et al., 2020): optimizing heating processes, selecting and formulating raw materials appropriately, and adding exogenous inhibitors. Among these, natural plant extracts have attracted increasing attention for their dual potential to enhance flavor and improve safety. Studies show that polyphenol-rich extracts from tea, turmeric, blueberry, acerola cherry, grape seed, and apple peel can effectively suppress HAAs formation (Dong, Ye, et al., 2024). Two main mechanisms may explain this effect: first, natural polyphenols can act as antioxidants, scavenging free radicals and blocking HAAs synthesis (Ma et al., 2025); second, they may trap reactive carbonyl species, thus inhibiting key intermediates reactions (Zhou et al., 2024). However, the efficacy of natural extracts depends strongly on polyphenol type, source and concentration. Some phenolic and flavonoid compounds, in particular, show limited inhibition against non-polar HAAs (Dong et al., 2020).

*Amomum villosum Lour*. (*A. villosum*), a perennial herb of the Zingiberaceae family, has significant ornamental, medicinal and culinary value. Its distribution mainly covers the tropical and subtropical regions of Asia, including China, India, Thailand, Vietnam and other Southeast Asian countries. This species is widely used in traditional Chinese medicine for its unique medicinal properties, such as dampness elimination, appetite enhancement, spleen warming, diarrhea prevention, qi regulation, and fetal stabilization (Feng et al., 2024). It is rich in volatile oils—primarily borneol acetate, camphor and borneol—as well as polyphenolic compounds such as quercetin and kaempferol derivatives, along with polysaccharides. These components contribute to multi-biological activities, including anti-inflammatory, antioxidant, antibacterial and gastrointestinal regulatory effects (Gao et al., 2023; Peng et al., 2025). In recent years, the notable therapeutic and nutritional value of *A. villosum* have attracted significant attention, leading to extensive and meaningful research by scholars. Currently, numerous bioactive compounds have been successfully isolated and identified, showcasing a diverse array of pharmacological activities and medicinal benefits (Feng et al., 2024). For example, recent studies report that extracts of *A. villosum* can effectively scavenge DPPH radicals, hydroxyl radicals, and superoxide anions in a dose-dependent manner (Zhang et al., 2024), indicating significant antioxidant potential. This property suggests that the extract may inhibit the formation of harmful free radical-mediated compounds generated during thermal processing. Despite its traditional uses and established bioactivity, the ability of *A. villosum* polyphenols to suppress HAAs formation in heat-processed meat products, and the mechanisms involved remains poorly studied. Therefore, the exploration and development of the inhibitory effect and mechanism of this plant on HAAs holds great research and development value.

Stricter environmental regulations have underscored the necessity for “green” extraction methods (Sahu et al., 2025). Deep eutectic solvents (DESs) can be entirely derived from natural substances (such as organic acids, carbohydrates, and amino acids), which endows them with remarkable environmental-friendly properties, including low toxicity, good biodegradability, and high biocompatibility (Wang et al., 2026). It has been demonstrated that DESs forms strong hydrogen bonds with polyphenols, thereby effectively disrupting intermolecular forces between polyphenol molecules (Sahu et al., 2025). In recent years, an increasing number of researchers have employed the DESs method to extract natural active substances. For instance, Yu et al. (2023) reported that the yield of tea saponins obtained through the DESs extraction process was 27% higher than that obtained using the ethanol method. Liu et al. (2022) used the ultrasonic-assisted DESs extraction technology to extract saponins from epimedium. They found that the extraction rate was significantly increased by 1.33–3.23 times compared with conventional solvents (water and 80% ethanol).

**Scheme 1:**
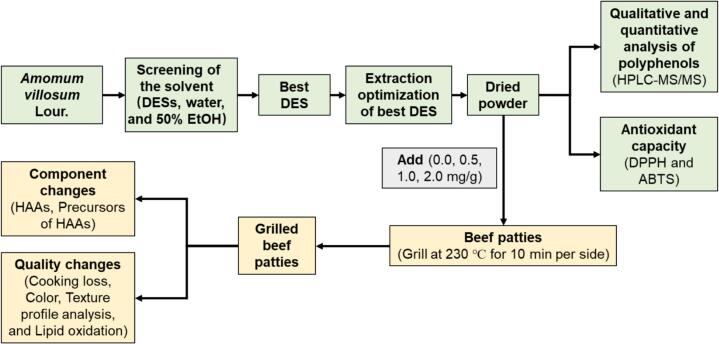
Schematic illustration.

This study establishes an efficient extraction protocol for *A. villosum* using ultrasound-assisted extraction with choline chloride-based DESs under optimized conditions, and then evaluated in grilled beef patties to assess its effect on the formation of 13 HAAs (Scheme 1). We further investigated how the extract influences key HAAs precursors—including glucose, creatine, creatinine, and free amino acids—as well as lipid oxidation during grilling. The antioxidant activity and phenolic profile of the extract were also characterized. Results clarify the HAAs inhibition properties of *A. villosum* and its potential underlying mechanisms. These insights offer a theoretical basis and practical strategy for developing natural, effective HAAs inhibitors, while supporting the use of *A. villosum* in functional seasonings and healthier meat products.

## 2 Materials and methods

### 2.1 Materials and reagents

Dried fruits of *A. villosum* were sourced from Guangzhou Baizhen Trading Co., Ltd. (Guangzhou, China), and fresh beef was procured from Guangzhou Changban Comprehensive Market (Guangzhou, China). A mixed standard solution of 13 HAAs, including 4,7,8-Trimethylimidazo[4,5-*f*]quinoxaline (4,7,8-TriMeIQx), 4,8-Dimethylimidazo[4,5-*f*]quinoxaline (4,8-DiMeIQx), 7,8-Dimethylimidazo[4,5-*f*]quinoxaline (7,8-DiMeIQx), 8-Methylimidazo[4,5-*f*]quinoxaline (8-MeIQx), 2-Amino-9H-pyrido[2,3-*b*]indole (AαC), 2-Aminodipyrido[1,2-a:3′,2′-d]imidazole (Glu-P-1), 2-Aminopyrido[1,2-a:3′,2′-d]imidazole (Glu-P-2), 1-Methyl-9H-pyrido[3,4-*b*]indole (Harman), Imidazo[4,5-*f*]quinoxaline (IQx), 2-Amino-3,4-dimethylimidazo[4,5-*f*]quinoline (MeIQ), 9H-Pyrido[3,4-*b*]indole (Norharman), 2-Amino-1-methyl-6-phenylimidazo[4,5-*b*]pyridine (PhIP), and 3-Amino-1,4-dimethyl-5H-pyrido[4,3-*b*]indole (Trp-P-1), was purchased from Alta Scientific Co., Ltd. (Tianjin, China). Acetonitrile and methanol (both HPLC grade) were obtained from Tianjin Damao Chemical Reagent Factory (Tianjin, China). The same supplier provided acetic acid and ammonium acetate. DPPH, ABTS, choline chloride, oxalic acid, protocatechuic acid, catechin, vanillic acid, epicatechin, and AB-8 macroporous adsorption resin were acquired from Shanghai Macklin Biochemical Co., Ltd. (Shanghai, China). All other reagents used were of analytical grade.

### 2.2 Preparation of *A. villosum* extract

#### 2.2.1 Preparation of crude extract of *A. villosum*

The dried fruits of *A. villosum* were ground into a fine powder using a high-speed blender (Model L6-L621A, Joyoung Co., Ltd., China) and stored in a desiccator. For extraction, 500 mg of the powdered sample was combined with 10 mL of solvent and heated in a water bath at 40 °C for 30 min. The mixture was then treated using an ultrasonic cleaner (SN-QX-100D, Sunne Co., Ltd., China) at 540 W for 40 min. After extraction, the solution was diluted with 30 mL of ultrapure water and coarsely filtered through gauze. The filtrate was centrifuged at 7000 r/min for 5 min, and the resulting supernatant was collected as the crude extract of *A. villosum*.

#### 2.2.2 Preparation and screening of DESs

DESs were prepared following previously reported methods with modifications (Gerçek et al., 2025; Yücel et al., 2025). Choline chloride served as the hydrogen bond acceptor (HBA), while various compounds—urea, glycerol, ethylene glycol, 1,2-propylene glycol, malic acid, lactic acid, 1,4-butanediol, citric acid, oxalic acid, and glucose—acted as hydrogen bond donors (HBD). The HBA and HBD were mixed at specific molar ratios (Table 1) and heated under continuous stirring in an 80 °C water bath until a clear, homogeneous liquid formed. Crude extracts of *A. villosum* were then obtained using these ten DESs, along with water and 50% ethanol as reference solvents. The total phenolic content of each extract was subsequently determined. Total phenolic content was assessed by the Folin-Ciocalteu method according to Zhang et al. (2014). A gallic acid standard curve was established by plotting absorbance against concentration, yielding the linear regression equation y = 0.0171× + 0.0743 with a coefficient of determination (R^2^) of 0.9978.

**Table 1:** The composition of ten kinds of deep eutectic solvents.

Group	HBA	HBD	Molar Ratio
DES-1	Choline chloride	1,4-Butanediol	1:2
DES-2		1,2-Propanediol	1:2
DES-3		Glucose	2:1
DES-4		Ethylene glycol	1:2
DES-5		Glycerol	1:2
DES-6		Urea	1:2
DES-7		Citric acid	1:1
DES-8		Malic acid	1:2
DES-9		Lactic acid	1:2
DES-10		Oxalic acid	1:1

Note: The “Molar Ratio” is meant to be the molar ratio between HBA and HBD. DES: Deep eutectic solvents, HBA: hydrogen bond acceptors, HBD: hydrogen bond donors (HBD).

#### 2.2.3 Other extraction optimization

Based on the selected optimal DESs, single-factor experiments were performed to optimize extraction conditions. The influence of key parameters on phenolic extraction yield from *A. villosum* was examined, including water content (10–60%), solid-to-liquid ratio (1:10 to 1:50), ultrasonication time (0–90 min), and heating temperature (50–90 °C). The total phenolic content of each extract was subsequently determined.

#### 2.2.4 Purification of *A. villosum* extract

Following process optimization, a scale-up extraction was conducted to prepare bulk crude extract for purification. The extract was loaded onto a pre-treated AB-8 macroporous resin column (2.6 cm × 60 cm) at a flow rate of 3 BV/h. Impurities were first eluted with ultrapure water at 4 BV/h, followed by target compound collection using 50% (*v*/v) ethanol at 2 BV/h. The ethanol eluate was concentrated by rotary evaporation at 40 °C, and the remaining aqueous phase was removed by freeze-drying to obtain the final purified *A. villosum* extract.

### 2.3 Preparation of grilled beef patties

Fresh ground beef was thawed and divided into four groups, supplemented with 0, 0.5, 1.0, or 2.0 mg/g of *A. villosum* extract, respectively. Each mixture was thoroughly blended and marinated at 4 °C for 4 h. Portions of 30.0 g from each group were then molded into patties using Petri dishes (6.0 cm in diameter, 1.5 cm in depth), with three replicates per group. Patties were baked in a preheated oven at 230 °C for 10 min per side. After cooking, samples were immediately analyzed for cooking loss, color, and texture. The remaining material was frozen, ground into a homogeneous powder, and stored at −20 °C for subsequent analysis of heterocyclic amines, amino acids, creatine, and glucose.

### 2.4 Extraction and determination of HAAs

HAAs were extracted and quantified according to the method outlined in NY/T 3904–2021. Briefly, 2.00 g of sample was added to 10 mL of water and shaken for 20 min. 10 mL of 1% acetic acid acetonitrile solution was added to the mixture and shaken for 15 min. Subsequently, 4.0 g of magnesium sulfate and 1.0 g of anhydrous sodium acetate were added and vortexed for 1 min. Centrifuge at 4 °C and 10,000 r/min for 10 min, and purify the upper organic phase (6 mL). Subsequently, 0.9 g of magnesium sulfate, 0.3 g of PSA, and 0.3 g of C18 filler were added and homogenized for 1 min, followed by centrifugation at 4 °C and 10,000 r/min for 5 min. 1.0 mL of supernatant was taken, dried it with nitrogen at 30 °C, dissolved in 0.50 mL of methanol, vortexed and mixed well, injected through a microporous membrane (Nylon 6, 0.22 μm, Jinteng, Tianjin, China) using a syringe, and transferred it to an injection bottle for testing.

Analysis was performed using UHPLC-ESI-QQQ-MS/MS (Agilent 1290–6470, USA). The separation was carried out on a ZORBAX Eclipse Plus C18 column (1.8 μm, 3.0 × 50 mm, Agilent, USA) at 30 °C, with mobile phases of 10 mmol/L ammonium acetate-acetic acid (A) and acetonitrile (B) at 0.4 mL/min. The injection volume was 2 μL. The gradient elution procedure: 0 min, 95% A + 5% B; 0.5 min, 95% A + 5% B; 5.0 min, 85% A + 15% B; 7.0 min, 73% A + 17% B; 8.0 min, 45% A + 55% B; 8.5 min, 73% A + 17% B; 9.0 min, 95% A + 5% B; 10 min, 95% A + 5% B. Mass spectrometry was performed in positive ESI mode with a scan range of *m/z* 100–500. Key parameters as follow: drying gas temperature, 300 °C; drying gas flow rate, 6 L/min; nebulizer gas pressure, 45 psi; sheath gas temperature, 250 °C; sheath gas flow rate, 10 L/min; capillary voltage, 4000 V; and nozzle voltage, 1000 V. Mass spectrometric detection was conducted in multi-reaction monitoring (MRM) scan mode. Data were processed using Mass Hunter MSC B.07.00 Build 30 software. The following 13 HAAs were quantified based on external standard calibration curves (**Table S1**): 4,7,8-TriMeIQx, 4,8-DiMeIQx, 7,8-DiMeIQx, 8-MeIQx, AαC, Glu-P-1, Glu-P-2, Harman, IQx, MeIQ, Norharman, PhIP, and Trp-P-1. A nine-point calibration curve was established at concentrations of 0.1, 0.5, 1, 5, 10, 50, 100, 500, and 1000 ng/mL.

### 2.5 Detection of creatine, creatinine, glucose and free amino acids

Creatine content was determined according to Cheng et al. (2019), with quantification based on a standard curve (y = 0.02247× + 0.11517, R^2^ = 0.9939).

Creatinine levels were measured using a creatinine assay kit (Nanjing Jiancheng Bioengineering Institute, Nanjing, China). In short, 1.0 g of the sample was mixed with 20 mL of 30 mg/mL trichloroacetic acid and homogenized for 1 min. 20 mL of the filtrate was collected, 4 mL of ether was added and shaken vigorously. Then, 6 μL of the lipid-free layer was taken out and followed the instructions of the kit for the determination.

1.0 g of sample was added to 20 mL of ultrapure water and homogenized for 1 min. Centrifuged the homogenate at 10000 rpm for 10 min, the supernatant was collected and analyzed according to the assay kit (Shanghai Beyotime Biological Co., Ltd., Shanghai, China), calibrated against a standard curve (y = 5.34167× + 0.03278, R^2^ = 0.9994).

1 g roasted beef sample was weighed and homogenized with 20 mL of 0.03 g/mL sulfosalicylic acid solution at 12,000 rpm for 25 s. The homogenate was centrifuged at 4000 rpm for 10 min at 4 °C. 5 mL of the supernatant was mixed with 2 mL of n-hexane, shaken thoroughly. The aqueous phase (1 mL) was filtered through a 0.22 μm membrane and analyzed. Free amino acid profiles were obtained using an automatic amino acid analyzer (Sykam, S—433D, Japan) following the procedure described by Zhang et al. (2023).

### 2.6 Determination of TBARS value

The TBARS value was measured following Xu et al. (2023). 1.0 g of sample was mixed with 5 mL of 7.5% (*w*/*v*) trichloroacetic acid. The mixture was stirred at 8000 rpm for 1 min in an ice bath, then centrifuged at 10000 rpm for 10 min at 4 °C. 0.1 mL of the supernatant was taken and analyzed it using a lipid oxidation (MDA) assay kit (Shanghai Beyotime Biological Co., Ltd., Shanghai, China). A standard curve (y = 0.01372× + 0.0405, R^2^ = 0.9980) was prepared using 1,1,3,3-tetra ethoxy propane, and the results were expressed as mg of MDA per kg of meat.

### 2.7 The antioxidant activity of *A. villosum* extract

Lyophilized *A. villosum* extract was dissolved in 50% ethanol to prepare test solutions at gradient concentrations. Ascorbic acid solutions at equivalent concentrations were used as the reference standard.

The DPPH radical scavenging activity was measured following Cheng et al. (2021). The absorbance value of the blank group denotes A_1_, the absorbance value of the sample group denotes A_2_, the absorbance value of 400 μL sample + 3200 μL water denotes A_3_. The DPPH radical scavenging activity was expressed as the scavenging rate, calculated using Eq. (1).

(1) $$\mathrm{DPPH radical scavenging activity}\left(\%\right)=\left(1-\frac{A2-A3}{A1}\right)\times 100$$

ABTS cation radical scavenging activity was assessed according to Szydłowska-Czerniak et al. (2025). The absorbance value of the blank group denotes A_1_, the absorbance value of the sample group denotes A_2_, the absorbance value of 400 μL sample + 3200 μL water denotes A_3_. The ABTS radical scavenging activity was expressed as the scavenging rate, calculated using Eq. (2).

(2) $$\mathrm{ABTS radical scavenging activity}\left(\%\right)=\left(1-\frac{A2-A3}{A1}\right)\times 100$$

### 2.8 Phenolic composition analysis of *A. villosum* extract

Phenolic compounds in the *A. villosum* extract were characterized using UHPLC-ESI-QTOF-MS/MS (Agilent 1290–6540, USA) following Zhang et al. (2024). The freeze-dried extract was dissolved in 50% chromatographic-grade methanol, filtered through a 0.22 μm syringe filter before analysis. The separation was performed on a ZORBAX Eclipse Plus C18 column (100 mm × 2.1 mm, 1.8 μm, Agilent, USA). Chromatographic conditions were as follows: flow rate, 0.3 mL/min; column temperature, 40 °C; injection volume, 10 μL. The mobile phase consisted of water (A) and acetonitrile (B), with the following gradient: 0 min, 99% A + 1% B; 4 min, 95% A + 5% B; 24 min, 10% A + 90% B; 25 min, 10% A + 90% B; 30 min, 1% A + 99% B. Quantification of major polyphenols—protocatechuic acid, catechin, vanillic acid, and epicatechin—was conducted with UHPLC-ESI-QQQ-MS/MS (Agilent 1290–6470, USA). Mass spectrometry was performed in negative ESI mode with a scan range of *m/z* 50–1000. Key parameters as follow: ion source gas and auxiliary gas, 55 psi; curtain gas, 40 psi; temperature, 550 °C; ion spray voltage, −4500 V; declustering potential, −80 V; collision energy, −35 eV; collision energy spread, 15 eV. Full-scan MS and data-dependent MS/MS scans were acquired. Data were processed using Mass Hunter MSC B.07.00 Build 30 software.

### 2.9 Cooking loss, color and texture profile analysis of grilled beef patties

Cooking loss was calculated for each beef patty by measuring its weight before and after grilling using Eq. (3).

(3) $$\text{Cooking loss}\;\left(\%\right)=\frac{\mathrm{Raw}\;\text{weight}\;\left(g\right)-\text{Cooked weight}\;\left(g\right)}{\mathrm{Raw}\;\text{weight}\;\left(g\right)}\times 100\%$$

Color parameters (L*, a*, b*) of the grilled patties were measured with a portable colorimeter (DS 200, FigSpec, Hangzhou, China) under standard illuminant D65 according to Liu et al. (2024). Three replicate measurements were taken for each sample.

Texture profile analysis was performed using a texture analyzer according to Li et al. (Li et al., 2020). Hardness, springiness, cohesiveness, chewiness, and resilience were evaluated.

### 2.10 Statistical analysis

Statistical analysis was performed using SPSS 16.0 (IBM Corp., USA). Univariate analysis of variance followed by Tukey's-b test was applied to assess intergroup differences, with significance defined at *P* < 0.05. All figures were prepared using Origin 2025 (OriginLab Corp., USA).

## 3 Results and discussion

### 3.1 Extraction optimization of *A. villosum* extract

**Fig. 1:**
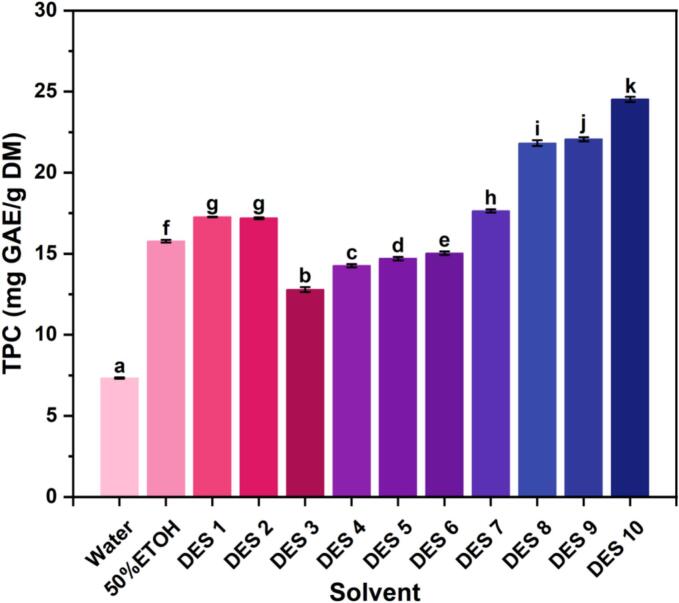
Effect of the different solvents on total phenolic content. All samples were extracted under same conditions: water content, 30%; solid-to-liquid ratio: 1:20; ultrasonication time, 40 min; and heating temperature, 40 °C.

This study utilized ultrasonic-assisted DESs extraction to isolate polyphenols from *A. villosum*. Ultrasound enhances the dissolution of active compounds by disrupting plant cell walls and generating cavitation effects (Chemat et al., 2017). DESs represent a class of green solvents characterized by low volatility, low toxicity, and cost efficiency. Their strong hydrogen-bonding capacity facilitates efficient polyphenol extraction (Lin et al., 2025). As shown in Fig. 1, under identical conditions, the extraction efficiency of *A. villosum* polyphenols varied significantly across solvents. DES 10 (choline chloride: oxalic acid = 1:1) yielded the highest polyphenol content (24.52 ± 0.16 mg GAE/g DM). Previous studies indicate that pH influences molecular interactions between DESs and target compounds (Duan et al., 2016). Oxalic acid in DES 10 may enhance extraction by disrupting cell walls via acid-catalysed hydrolysis, converting bound polyphenols to free forms, and inhibiting polyphenol oxidase (Cui et al., 2023). Its low molecular weight (90.03 g/mol) also reduces solvent viscosity, improving penetration into plant tissues. Moreover, the two carboxyl groups in oxalic acid provide a high density of hydrogen-bonding sites, strengthening polyphenol solubility.

**Fig. 2:**
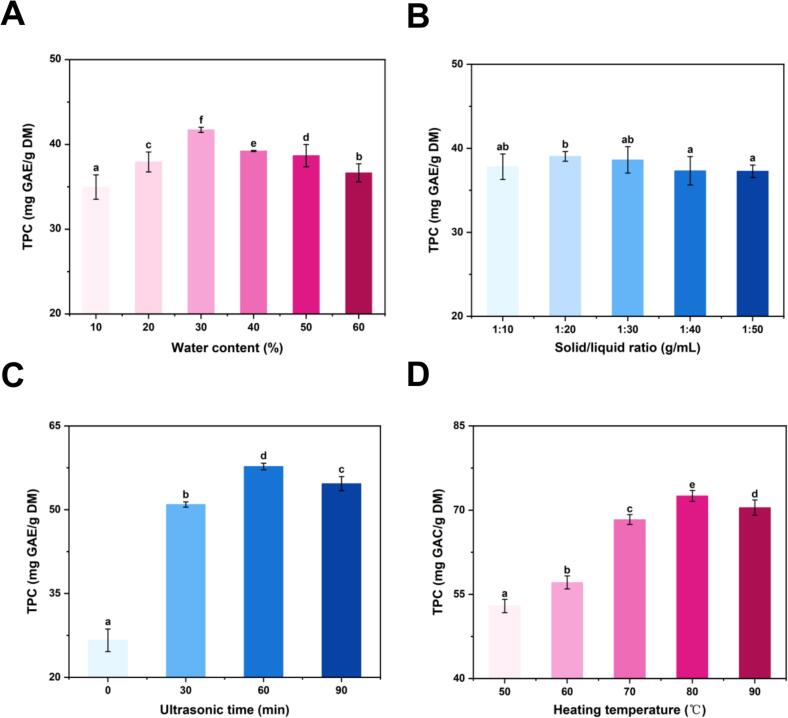
(A) Effect of the water content on total phenolic content. (B) Effect of the solid/liquid ratio on total phenolic content. (C) Effect of the ultrasonic time on total phenolic content. (D) Effect of the heating temperature on total phenolic content. All samples were extracted used the DES 10.

Based on these results, DES 10 was selected for further optimization of water content, solid/liquid ratio, ultrasonication time and heating temperature (Fig. 2). The optimal conditions were identified as 30% (*w*/w) water content, a solid/liquid ratio of 1:20 g/mL, 60 min of ultrasonication, and 80 °C heating temperature, yielding 72.53 ± 0.97 mg GAE/g DM of polyphenols. This outcome can be attributed to several factors: excessive water increases DES viscosity, while insufficient water disrupts the hydrogen-bonding network between HBA and HBD (Peng et al., 2025). A low solid-to-liquid ratio limits sample-solvent contact, whereas a high ratio wastes solvent. Similarly, insufficient ultrasonication time or temperature restricts compound diffusion, while excessive conditions may degrade polyphenols (Hu et al., 2024).

### 3.2 Inhibitory effect of *A. villosum* extract on HAAs

HAAs content in grilled beef patties was analyzed by UHPLC-ESI-QQQ-MS/MS. As summarized in Table 2, 13 HAAs were detected in the control group. Non-polar HAAs (Harman, Norharman, Glu-P-1, Glu-P-2, AαC, and Trp-P-1) accounted for 57.89% of total HAAs. Total HAA levels measured 303.50 ± 56.35, 172.64 ± 16.78, 220.95 ± 27.70, and 268.93 ± 32.57 ng/100 g in patties supplemented with 0, 0.5, 1.0, and 2.0 mg/g of *A. villosum* extract, respectively. The highest inhibition rate (43.11%) was achieved at 0.5 mg/g. The addition of *A. villosum* extract significantly reduced the levels of nine HAAs (4,7,8-TriMeIQx, 4,8-DiMeIQx, 7,8-DiMeIQx, 8-MeIQx, AαC, IQx, MeIQ, PhIP, and Trp-P-1), compared to the control, with inhibition rates ranging from 21.67% to 100%. Notably, no significant differences in inhibition were observed among the three extract concentrations, suggesting maximal suppression occurred even at the lowest dose. This aligns with reports that polyphenol-rich extracts from ginger and citrus peel prepared using DESs similarly reduce HAAs formation in thermally processed meat (Xu et al., 2022). In contrast, Harman and Norharman levels increased significantly at higher extract concentrations. A similar phenomenon was reported by Zeng et al. (2018) with *Zanthoxylum bungeanum* extract and Yan et al. (2022) with soybean protein isolate, where elevated doses promoted the formation of these two HAAs. *A. villosum* extract contain protocatechuic acid (Zhang, Shuai, et al., 2024), some research pointed out that protocatechuic acid has a synergistic effect on the formation of Harman and Norharman, which at higher concentrations may facilitate their generation during grilling (Zeng et al., 2016).

**Table 2:** The inhibitory effect of *A. villosum* extract on HAAs in grilled beef patties.

Content (ng/100 g)	Group			
	0.0	0.5	1.0	2.0
4,7,8-TriMeIQx	12.90 ± 11.56^b^	1.44 ± 2.07^a^	0.34 ± 0.58^a^	0.00 ± 0.00^a^
4,8-DiMeIQx	12.34 ± 5.49^b^	3.27 ± 0.54^a^	3.49 ± 0.49^a^	2.59 ± 0.71^a^
7,8-DiMeIQx	17.50 ± 5.64^b^	9.37 ± 0.43^a^	9.36 ± 0.68^a^	8.53 ± 0.72^a^
8-MeIQx	22.67 ± 6.23^b^	10.53 ± 0.87^a^	9.97 ± 3.62^a^	13.11 ± 5.48^a^
AαC	40.90 ± 22.23^b^	13.82 ± 4.45^a^	7.54 ± 2.22^a^	3.98 ± 1.29^a^
Glu-P-1	12.11 ± 6.60^a^	9.25 ± 1.28^a^	8.52 ± 0.32^a^	7.97 ± 0.72^a^
Glu-P-2	8.62 ± 0.92^a^	7.72 ± 0.78^a^	8.20 ± 1.49^a^	7.76 ± 0.22^a^
Harman	37.27 ± 17.94^a^	37.83 ± 1.92^a^	77.07 ± 12.70^b^	117.24 ± 16.97^c^
IQx	18.20 ± 2.33^b^	14.26 ± 1.08^a^	13.66 ± 0.65^a^	13.53 ± 1.03^a^
MeIQ	17.80 ± 8.86^b^	4.77 ± 1.50^a^	3.40 ± 0.97^a^	3.53 ± 0.86^a^
Norharman	47.12 ± 7.08^ab^	37.18 ± 3.90^a^	55.73 ± 9.59^b^	72.40 ± 10.19^c^
PhIP	26.40 ± 6.21^b^	11.34 ± 1.07^a^	12.72 ± 1.36^a^	9.48 ± 3.17^a^
Trp-P-1	29.67 ± 18.03^b^	11.86 ± 1.29^a^	10.96 ± 2.69^a^	8.81 ± 1.35^a^
Total	303.50 ± 56.35^c^	172.64 ± 16.78^a^	220.95 ± 27.70^ab^	268.93 ± 32.57^bc^

Note: Values are given as mean ± standard deviation (*n* = 3). Different letters in the same column indicate significant difference (*p* < 0.05).

### 3.3 Change of creatine, creatinine, glucose and free amino acid in grilled beef patties

**Fig. 3:**
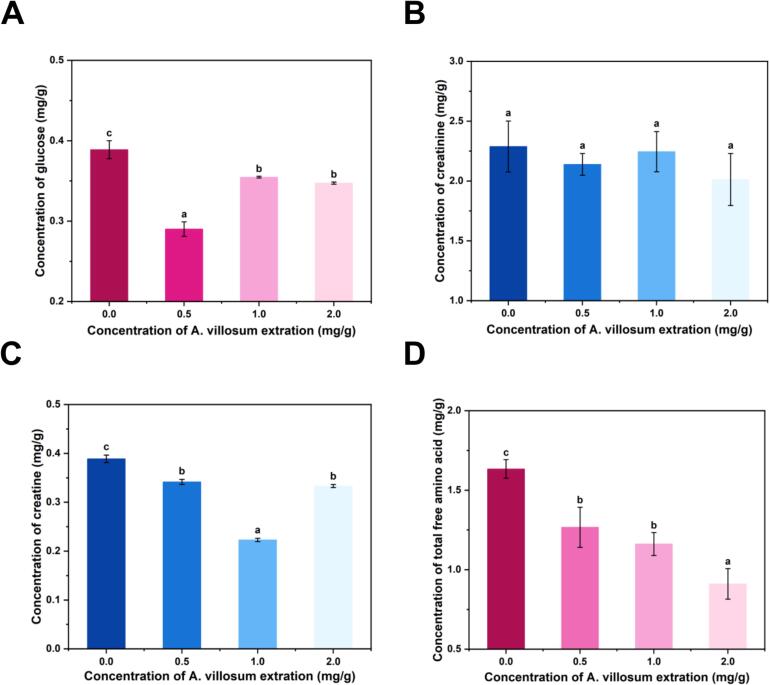
The effect of *A. villosum* extract on precursors in grilled beef patties. (A) Glucose. (B) Creatinine. (C) Creatine. (D) Total free amino acid.

Free amino acids, glucose, creatine, and creatinine serve as key precursors in the formation of HAAs, with their concentrations shifting during the grilling process. As shown in Fig. 3, the addition of *A. villosum* extract significantly reduced glucose and creatine levels in grilled beef patties compared to the control, while creatinine content remained unchanged. Studies have shown that glucose is closely associated with the formation of IQ-type and IQx-type HAAs, while creatine strongly correlates with PhIP, IQ-type, and IQx-type HAAs (Zhang et al., 2024). In this study, increasing concentrations of *A. villosum* extract corresponded with decreased levels of PhIP, IQ-type and IQx-type HAAs. These results suggest that the extract may suppress HAA formation by lowering the availability of glucose and creatine. One the one hand, glucose could combine with phenolic acids to form novel glycoside compounds (Nam et al., 2017). One the other hand, under heating conditions, the phenolic hydroxyl groups of polyphenols may oxidize to form quinones. These quinones can form covalent bonds with the amino groups or mercapto groups of amino acids, forming polymers or insoluble complexes. This process leads to a reduction in free amino acids (Zhang, Huang, et al., 2024). Moreover, the study also found that polyphenols (especially those with catechol or meta-catechol structures) are easily oxidized into quinones under heating and in the presence of oxygen. Quinone compounds may undergo Michael addition reactions or Schiff base reactions (Nan & Liu, 2005) with the amino groups of creatine, forming polyphenol-creatine adducts, which reduce the creatine content in grilled beef patties.

The content of 17 free amino acids in the grilled patties is summarized in Table 3. All treatment groups showed a dose-dependent decrease in free amino acids relative to the control. Since free amino acids are essential precursors for HAA formation. For example, glycine, threonine, alanine, lysine, and serine have been demonstrated to promote MeIQx-type HAAs (Margaretha & Kerstin, 1991). Therefore, the observed reduction suggests that *A. villosum* extract limits HAA formation by depleting these key reactants.

**Table 3:** The effect of *A. villosum* extract on free amino acid in grilled beef patties (n = 3).

Free amino acid (mg/g)	Group			
	0.0	0.5	1.0	2.0
Asp	0.032 ± 0.002^c^	0.031 ± 0.004^c^	0.021 ± 0.008^b^	0.011 ± 0.001^a^
Thr	0.069 ± 0.008^c^	0.054 ± 0.004^b^	0.038 ± 0.002^a^	0.027 ± 0.003^a^
Ser	0.082 ± 0.005^c^	0.066 ± 0.007^b^	0.055 ± 0.002^b^	0.041 ± 0.005^a^
Glu	0.071 ± 0.013^c^	0.069 ± 0.009^bc^	0.047 ± 0.003^ab^	0.040 ± 0.011^a^
Gly	0.170 ± 0.016^d^	0.140 ± 0.014^c^	0.100 ± 0.012^b^	0.078 ± 0.010^ab^
Ala	0.518 ± 0.055^c^	0.396 ± 0.041^b^	0.394 ± 0.033^b^	0.312 ± 0.026^ab^
Cys	0.001 ± 0.001^a^	0.002 ± 0.001^a^	0.004 ± 0.000^b^	0.004 ± 0.001^b^
Val	0.075 ± 0.005^c^	0.056 ± 0.004^b^	0.059 ± 0.009^b^	0.045 ± 0.004^ab^
Met	0.042 ± 0.007^c^	0.030 ± 0.002^ab^	0.034 ± 0.001^bc^	0.031 ± 0.001^ab^
Ile	0.062 ± 0.007^d^	0.042 ± 0.004^bc^	0.047 ± 0.003^c^	0.037 ± 0.005^b^
Leu	0.100 ± 0.016^b^	0.071 ± 0.008^a^	0.086 ± 0.006^ab^	0.067 ± 0.007^a^
Tyr	0.052 ± 0.002^c^	0.035 ± 0.008^b^	0.039 ± 0.005^b^	0.030 ± 0.004^ab^
Phe	0.062 ± 0.002^b^	0.043 ± 0.002^a^	0.050 ± 0.007^ab^	0.040 ± 0.012^a^
His	0.060 ± 0.007^b^	0.052 ± 0.005^b^	0.042 ± 0.003^a^	0.033 ± 0.005^a^
Lys	0.080 ± 0.006^c^	0.0610 ± 0.006^b^	0.054 ± 0.003^b^	0.042 ± 0.005^a^
Arg	0.110 ± 0.016^d^	0.086 ± 0.011^c^	0.065 ± 0.003^b^	0.050 ± 0.008^ab^
Pro	0.048 ± 0.004^c^	0.034 ± 0.004^b^	0.029 ± 0.001^ab^	0.021 ± 0.001^a^
Total	1.634 ± 0.058^c^	1.267 ± 0.126^b^	1.162 ± 0.072^b^	0.911 ± 0.096^a^

Note: Values are given as mean ± standard deviation (*n* = 3). Different letters in the same column indicate significant difference (*p* < 0.05).

### 3.4 Change of lipid oxidation in grilled beef patties

**Fig. 4:**
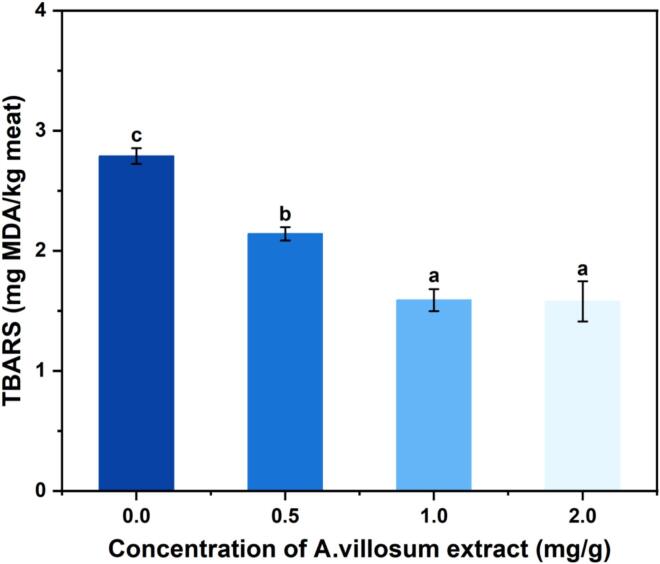
TBARS value (mg MDA/kg meat) of grilled beef patties (*n* = 3).

Lipid oxidation in grilled beef patties was assessed using TBARS. As illustrated in Fig. 4, TBARS values decreased significantly with increasing concentrations of *A. villosum* extract, indicating a concentration-dependent antioxidant effect. The lowest values were observed at 1.0 and 2.0 mg/g, beyond which no further reduction occurred. Free radicals generated during lipid oxidation play a key role in HAAs formation, and studies have shown a positive correlation between lipid oxidation products and HAA levels (Hwang & Ngadi, 2002). The observed reduction in TBARS suggests that *A. villosum* extract suppresses HAA formation by scavenging free radicals and limiting the production of lipid-derived HAA precursors. The inhibitory effect on lipid oxidation plateaued at high concentrations of *A. villosum* extract, corresponding to a stabilization in HAAs suppression.

### 3.5 Antioxidant capacity of *A. villosum* extract

**Fig. 5:**
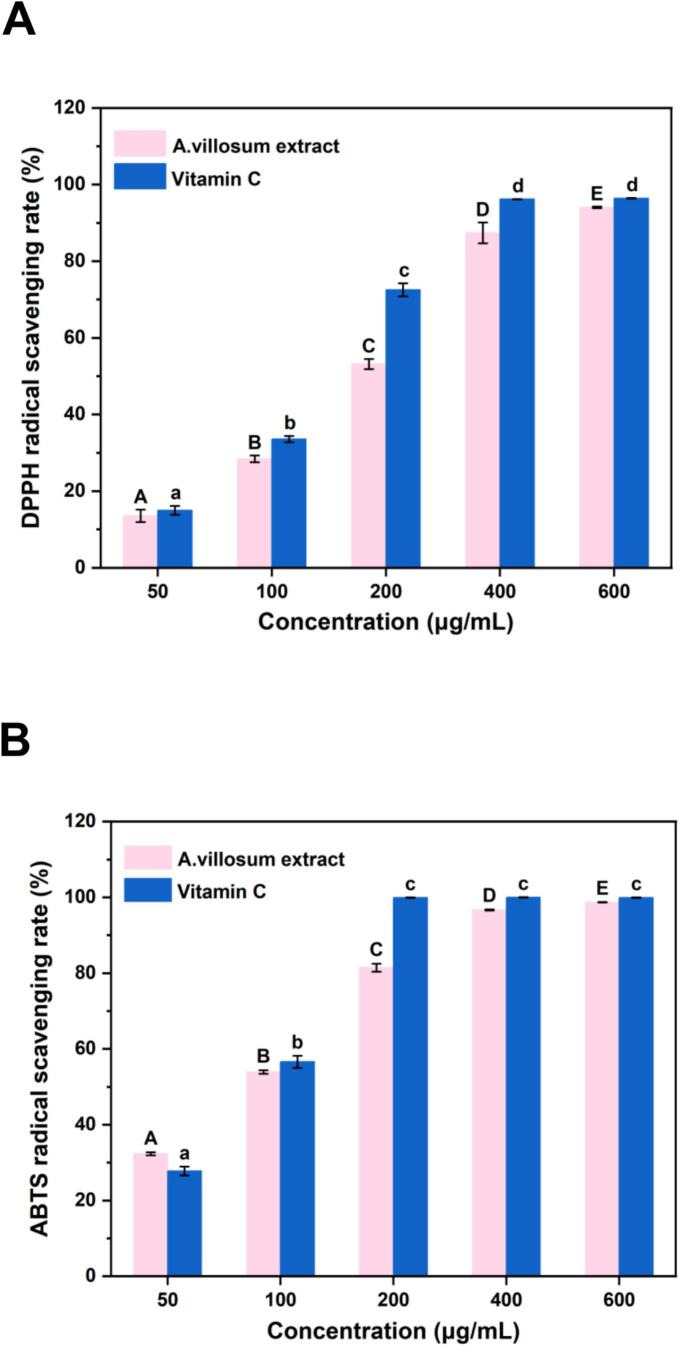
(A) DPPH radical scavenging rate of different concentrations of *A. villosum* extract and vitamin C. (B) ABTS radical scavenging rate of different concentrations of *A. villosum* extract and vitamin C.

The antioxidant activity of *A. villosum* extract was evaluated using DPPH and ABTS free radical scavenging assays, with vitamin C as a reference. As shown in Fig. 5, both DPPH and ABTS scavenging capacities increased in a concentration-dependent manner. The IC_50_ values for DPPH scavenging were 37.93 μg/mL for the extract and 39.97 μg/mL for vitamin C, while those for ABTS were 33.37 μg/mL and 32.49 μg/mL, respectively, indicating comparable antioxidant capacity. Notably, *A. villosum* extract significantly inhibited all five IQx-types HAAs analyzed. Since pyrazine is a key intermediate in IQx-type HAAs formation, and polyphenols can quench pyrazine cation radicals, the observed suppression is likely attributable to the radical-scavenging activity of the extract (Sakač et al., 2018).

### 3.6 Polyphenol composition in *A. villosum* extract

The phenolic profile of the *A. villosum* extract were analyzed by UHPLC-TOF-MS/MS, revealing 19 phenolic compounds (Table 4). Quantitative analysis using UHPLC-QQQ-MS/MS showed that protocatechuic acid and vanillic acid were the most abundant, at 458.32 ± 0.23 and 171.73 ± 0.12 mg/100 g, respectively (Table 5). These compounds, along with other identified phenolic acids and procyanidins, contribute significantly to the extract's strong antioxidant capacity (Kaur et al., 2022). The high procyanidin content also explains the extract's reddish-brown color and its mild effect on the appearance of grilled patties.

**Table 4:** Qualitative analysis of polyphenols in the extract of *A. villosum*.

Compounds	Formula	RT(min)	Mass	Mode	Significant Ion Mz
Citric acid	C_6_H_8_O_7_	1.185	192.0270	[M-H]^−^	191.0193
Protocatechuic acid	C_7_H_6_O_4_	2.889	154.0261	[M-H]^−^	153.0189
Vanillic acid hexoside	C_14_H_18_O_9_	4.113	330.0940	[M-H]^−^	329.0867
Vanillic acid hexoside isomer	C_14_H_18_O_9_	4.717	330.0943	[M-H]^−^	329.0870
Procyanidin B	C_30_H_26_O_12_	5.172	578.1410	[M-H]^−^	577.1339
Catechin	C_15_H_14_O_6_	5.246	290.0783	[M-H]^−^	289.0710
Vanillic acid	C_8_H_8_O_4_	5.420	168.0411	[M-H]^−^	167.0345
Procyanidin C	C_45_H_38_O_18_	5.519	866.2043	[M-H]^−^	865.1978
Procyanidin B isomer 1	C_30_H_26_O_12_	5.842	578.1408	[M-H]^−^	577.1335
Epicatechin	C_15_H_14_O_6_	5.966	290.0781	[M-H]^−^	289.0709
Caffeic acid	C_9_H_8_O_4_	6.189	180.0417	[M-H]^−^	179.0344
*p*-Coumaric acid	C_9_H_8_O_3_	6.810	164.0471	[M-H]^−^	163.0398
Procyanidin A	C_30_H_24_O_12_	6.992	576.1260	[M-H]^−^	575.1182
Ferulic acid	C_10_H_10_O_4_	7.281	194.0533	[M-H]^−^	193.0501
Procyanidin B isomer 2	C_30_H_26_O_12_	7.347	578.1402	[M-H]^−^	577.1333
Hyperoside	C_21_H_20_O_12_	7.604	464.0940	[M-H]^−^	463.0866
Syringaldehyde	C_9_H_10_O_4_	7.645	182.0575	[M-H]^−^	181.0502
Quercetin	C_15_H_10_O_7_	9.754	302.0419	[M-H]^−^	301.0346
9-Hydroxy-10,12-octadecadienoic acid (9-HODE)	C_18_H_32_O_3_	14.998	296.2340	[M-H]^−^	295.2269

**Table 5:** Quantitative analysis of polyphenols in the extract of *A. villosum*.

Compounds	Formula	RT (min)	Content (mg/100 g)
Protocatechuic acid	C_7_H_6_O_4_	2.889	458.32 ± 0.23
Catechin	C_15_H_14_O_6_	5.246	18.02 ± 0.07
Vanillic acid	C_8_H_8_O_4_	5.420	171.73 ± 0.12
Epicatechin	C_15_H_14_O_6_	5.966	19.38 ± 0.13

Catechin and epicatechin were present at 18.02 ± 0.07 and 19.38 ± 0.13 mg/100 g, respectively. As catechol-type polyphenols, they may inhibit PhIP formation by trapping phenylacetaldehyde—a key PhIP precursor—through electrophilic adduct formation (Dong, Chen, et al., 2024). This mechanism has been similarly reported for mangiferin, which contains an analogous catechol structure (Wang et al., 2021). During grilling, catechin and epicatechin likely react with phenylacetaldehyde to form stable adducts, thereby reducing the availability of intermediates needed for HAA generation.

The diversity of polyphenols in *A. villosum* supports its ability to suppress HAAs through multiple pathways, consistent with earlier reports on its phytochemical richness (Zhang et al., 2024; Su et al., 2025).

### 3.7 Quality changes of grilled beef patties

Cooking loss and color are key quality attributes of meat products, with the former influencing juiciness and the latter affecting consumer acceptance (Fan et al., 2022; Wang et al., 2025). As shown in Table 6, the addition of *A. villosum* extract had no significant impact on cooking loss or b* values compared to the control. However, a dose-dependent decrease in L* was observed, declining from 49.77 ± 3.15 in the control to 46.37 ± 3.34 at 2.0 mg/g. Conversely, a* values increased from 3.98 ± 0.99 in the control to 5.10 ± 0.69 and 5.44 ± 1.11 at 0.5 and 1.0 mg/g extract, respectively. These color modifications are likely attributable to natural pigments present in the extract.

**Table 6:** The influence of *A. villosum* extract on the cooking loss and color value of grilled beef patties.

Index		Group			
		0.0	0.5	1.0	2.0
Cooking loss(%)		48.71 ± 0.08^a^	51.02 ± 2.30^a^	51.44 ± 1.97^a^	48.55 ± 0.71^a^
Color	L*	49.77 ± 3.15^b^	48.14 ± 1.51^ab^	47.72 ± 1.83^ab^	46.37 ± 3.34^a^
	a*	3.98 ± 0.99^ab^	5.10 ± 0.69^bc^	5.44 ± 1.11^c^	3.74 ± 1.26^a^
	b*	9.10 ± 3.01^a^	10.99 ± 2.21^a^	9.08 ± 1.26^a^	8.70 ± 2.70^a^

Note: Values are given as mean ± standard deviation (*n* = 3). Different letters in the same column indicate significant difference (*p* < 0.05).

**Fig. 6:**
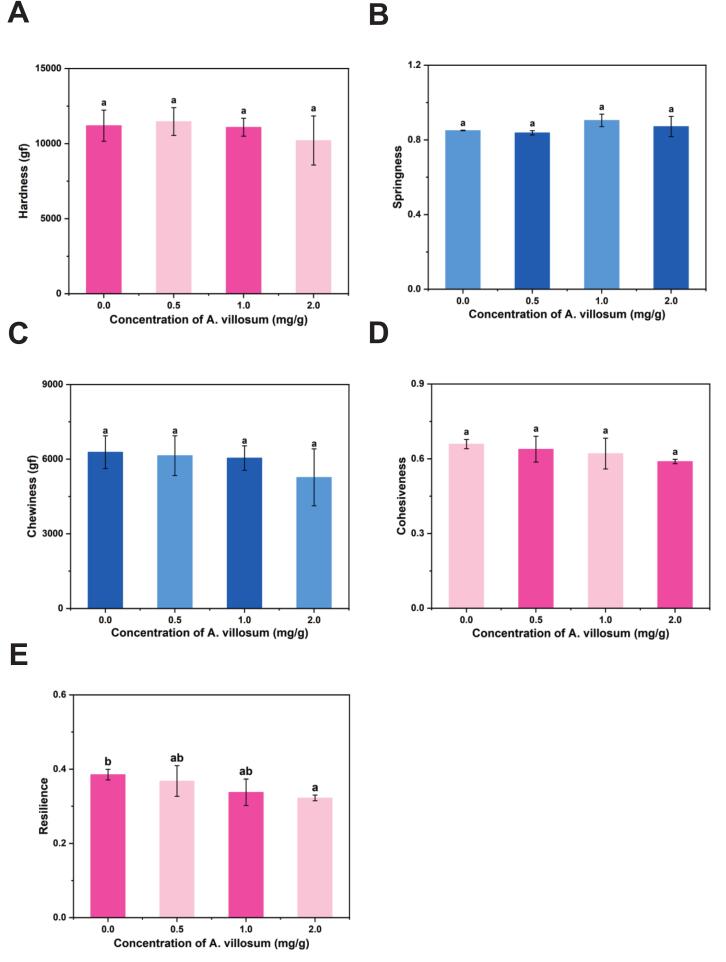
The influence of *A. villosum* extract on the texture properties of grilled beef patties. (A) Hardness. (B) Springiness. (C) Chewiness. (D) Cohesiveness. (E) Resilience.

Texture profile analysis revealed that extract incorporation did not significantly alter hardness, springiness, chewiness, or cohesiveness at any concentration (Fig. 6). Resilience remained unaffected except at 2.0 mg/g, where a slight but significant decrease was observed, possibly due to structural interference caused by the high polyphenol content.

## 4 Conclusion

In conclusion, this study established an efficient extraction protocol for *A. villosum* using ultrasonic-assisted DES (choline chloride: oxalic acid = 1:1), yielding 72.53 ± 0.97 mg GAE/g DM of polyphenols. The extract significantly reduced the levels of multiple HAAs and their precursors in grilled beef patties, with the highest total HAAs inhibition (43.11%) achieved at 0.5 mg/g. Two principal mechanisms are proposed: the potent antioxidant capacity and the adduction of phenolic compound with reactive HAA intermediates. It had no impact on quality attributes. These findings support the use of *A. villosum* as a natural HAAs inhibitor and contribute to its potential application in healthier grilled meat products. Adding natural plant extracts can effectively inhibit the formation of HAAs. However, exploring the optimal addition level of *A. villosum* extract in different foods and delving deeper into the mechanism remain issues that need to be addressed in the future. Additionally, protecting the activity of *A. villosum* polyphenols extract is also a direction for future research.

## CRediT authorship contribution statement

**Yuhao Huang:** Writing – original draft, Validation, Methodology, Investigation, Data curation. **Yanan Liu:** Writing – review & editing, Methodology, Data curation, Conceptualization. **Bao-Zhu Jia:** Methodology, Data curation, Conceptualization. **Zhen-Lin Xu:** Writing – review & editing, Supervision, Resources. **Lin Luo:** Writing – review & editing, Supervision, Resources, Funding acquisition.

## Declaration of competing interest

The authors declare that they have no known competing financial interests or personal relationships that could have appeared to influence the work reported in this paper.

## Data Availability

Data will be made available on request.

## References

[bb0005] Chemat F., Rombaut N., Sicaire A.G., Meullemiestre A., Fabiano-Tixier A.S., Abert-Vian M. (2017). Ultrasound assisted extraction of food and natural products. Mechanisms, techniques, combinations, protocols and applications. A review. Ultrasonics Sonochemistry.

[bb0010] Cheng Y., Yao M., Zhu Z., Dong X., Ali Khan I., Huang J., Zhou X., Huang M., Zhou G. (2019). Content, causes and analysis of heterocyclic amines in Chinese traditional braised chicken. Food Additives and Contaminants - Part A Chemistry, Analysis, Control, Exposure and Risk Assessment.

[bb0015] Cheng Y., Yu Y., Wang C., Zhu Z., Huang M. (2021). Inhibitory effect of sugarcane (Saccharum officinarum L.) molasses extract on the formation of heterocyclic amines in deep-fried chicken wings. Food Control.

[bb0020] Cui H., Chen F., Yu J., Hayat K., Zhang X. (2023). Accurate pH decrease induced inactivation of myrosinase and polyphenol oxidase in salted radish for color improvement. Food Bioscience.

[bb0025] Davis C.D., Snyderwine E.G. (1995). Protective effect of N-acetylcysteine against heterocyclic amine-induced cardiotoxicity in cultured myocytes and in rats. Food and Chemical Toxicology.

[bb0030] Ding Y., Li G., Zheng X., Yu M., Wen R., Hu Y. (2025). Inhibitory effects of hydrocolloids on protein-bound heterocyclic aromatic amine formation in fried chicken drumsticks and chemical model system : Insights from molecular docking analysis. Food Chemistry.

[bb0035] Dong H., Chen Q., Xu Y., Li C., Bai W., Zeng X., Wu Q., Xu H., Deng J. (2024). Effect and mechanism of polyphenols containing m-dihydroxyl structure on 2-amino-1-methyl-6-phenylimidazole [4, 5-b] pyridine (PhIP) formation in chemical models and roast pork patties. Food Chemistry: X.

[bb0040] Dong H., Xian Y., Li H., Bai W., Zeng X. (2020). Potential carcinogenic heterocyclic aromatic amines (HAAs) in foodstuffs: Formation, extraction, analytical methods, and mitigation strategies. Comprehensive Reviews in Food Science and Food Safety.

[bb0045] Dong H., Ye H., Bai W., Zeng X., Wu Q. (2024). A comprehensive review of structure–activity relationships and effect mechanisms of polyphenols on heterocyclic aromatic amines formation in thermal-processed food. Comprehensive Reviews in Food Science and Food Safety.

[bb0050] Duan L., Dou L.L., Guo L., Li P., Liu E.H. (2016). Comprehensive evaluation of deep eutectic solvents in extraction of bioactive natural products. ACS Sustainable Chemistry and Engineering.

[bb0055] Fan L., Ruan D., Shen J., Hu Z., Liu C., Chen X., Xia W., Xu Y. (2022). The role of water and oil migration in juiciness loss of stuffed fish ball with the fillings of pig fat/meat as affected by freeze-thaw cycles and cooking process. LWT- Food Science and Technology.

[bb0060] Feng L., Wang Z., Lei Z., Zhang X., Zhai B., Sun J., Guo D., Wang D., Luan F., Zou J., Shi Y. (2024). Amomum villosum Lour.: An insight into ethnopharmacological, phytochemical, and pharmacological overview. Journal of Ethnopharmacology.

[bb0065] Fuccelli R., Rosignoli P., Servili M., Veneziani G., Taticchi A., Fabiani R. (2018). Genotoxicity of heterocyclic amines (HCAs) on freshly isolated human peripheral blood mononuclear cells (PBMC) and prevention by phenolic extracts derived from olive, olive oil and olive leaves. Food and Chemical Toxicology.

[bb0070] Gao C., Zhu M., Xu W., Wang Y., Xiong L., Sun D., Sun M., Lin Y., Li H., Chen L. (2023). Chemical constituents from the stems and leaves of Amomum villosum Lour. And their anti-inflammatory and antioxidant activities. Bioorganic Chemistry.

[bb0075] Gerçek Y.C., Kutlu N., Çelik S., Bayram S., Kırkıncı S., Ecem Bayram N. (2025). Optimized ultrasonic-NaDES extraction of anthocyanins, polyphenolics, and organic acids from chokeberry fruit with blueness and antimicrobial evaluation. Microchemical Journal.

[bb0080] Gibis M. (2016). Heterocyclic aromatic amines in cooked meat products: Causes, formation, occurrence, and risk assessment. Comprehensive Reviews in Food Science and Food Safety.

[bb0085] Hu M., Han B., Xie L., Lu B., Bai D., Shi N., Liao Y., Wang Y., Liu L., Wu S., Lan R., Lei X., Shi C., Huang D., Li Y., Lin L., Zhang J. (2024). Ultrasonic assisted natural deep eutectic solvents as a green and efficient approach for extraction of hydroxytyrosol from olive leaves. Industrial Chemistry & Materials.

[bb0090] Hwang D.K., Ngadi M. (2002). Kinetics of heterocyclic amines formation in meat emulsion at different fat contents. Lebensmittel-Wissenschaft Und-Technologie.

[bb0095] Kaur J., Gulati M., Singh S.K., Kuppusamy G., Kapoor B., Mishra V., Corrie L. (2022). Discovering multifaceted role of vanillic acid beyond flavours: Nutraceutical and therapeutic potential. Trends in Food Science and Technology.

[bb0100] Kim H.S., Hur S.J. (2018). Changes in the mutagenicity of heterocyclic amines, nitrite, and N-nitroso compound in pork patties during in vitro human digestion. LWT - Food Science and Technology.

[bb0105] Lawana V., Um S.Y., Rochet J.C., Turesky R.J., Shannahan J.H., Cannon J.R. (2020). Neuromelanin modulates heterocyclic aromatic amine-induced dopaminergic neurotoxicity. Toxicological Sciences.

[bb0110] Li Y., He J., Quan W., He Z., Qin F., Tao G., Wang Z., Zeng M., Chen J. (2020). Effects of polyphosphates and sodium chloride on heterocyclic amines in roasted beef patties as revealed by UPLC-MS/MS. Food Chemistry.

[bb0115] Lin X., Zhou S., Sun Z., Cao M., Zhou T., Zhao L., Chen G. (2025). Deep eutectic solvent-based ultrasonic-assisted extraction of polyphenol from Chenopodium quinoa Willd.: Optimization and lipid-lowering activity. Food Chemistry.

[bb0120] Liu J., Feng C., Li Y., Zhang Y., Liang Q., Xu S. (2022). Photocatalytic detoxification of hazardous pymetrozine pesticide over two-dimensional covalent-organic frameworks coupling with. Separation and Purification Technology.

[bb0125] Liu Y., Chen J., Li H., Wang Y. (2024). Nanocomplexes film composed of gallic acid loaded ovalbumin/chitosan nanoparticles and pectin with excellent antibacterial activity: Preparation, characterization and application in coating preservation of salmon fillets. International Journal of Biological Macromolecules.

[bb0130] Ma Y., Zhou Y., Jiang X., Duan H., Ma Z., Ma Q., Li Z., Wang S. (2025). Multi-pathway inhibition of heterocyclic amines in braised chicken by carnosic acid: Combined experimental and DFT calculations. Food Chemistry.

[bb0135] Margaretha J., Kerstin S. (1991). Nutritional and Toxicological Consequences of Food Processing.

[bb0140] Meurillon M., Engel E. (2016). Mitigation strategies to reduce the impact of heterocyclic aromatic amines in proteinaceous foods. Trends in Food Science and Technology.

[bb0145] Nam S., Ko J., Jun W., Wee Y., Walsh M.K., Yang K., Kim D. (2017). Enzymatic synthesis of chlorogenic acid glucoside using dextransucrase and its physical and functional properties. Enzyme and Microbial Technology.

[bb0150] Nan G., Liu D. (2005). Elementary introduction to where Schiff base, Schiff base metal complexes derived from, reactive mechanism ways of synthesis and its prospect. Journal of ILi Teachers College.

[bb0155] Peng Z., Wang Y., Li W., Zhan B., Zhu L., Yang D., Li G., Zhang L., Zhao Z. (2025). Ultrasonic-assisted extraction of flavonoids from Amomum villosum Lour. Using natural deep eutectic solvent: Process optimization, comparison, identification, and bioactivity. Ultrasonics Sonochemistry.

[bb0160] Sahu S., Kumari D., Kuila A., Singh R., Sharma K., Verma R. (2025). Deep eutectic solvent extraction of polyphenol from plant materials: Current status and future prospects in food applications. Food Chemistry.

[bb0165] Sakač M., Đilas S., Jovanov P. (2018). The influence of polyphenols on the formation of free radicals detected in maillard reaction model systems. Food and Feed Research.

[bb0170] Su M., Jin R., Zhu J., Pei J., Wang Y., Chai X., Jiang M. (2025). Composition and antioxidant activity of flavonoids from two different species of Amomi Fructus extracted using natural deep eutectic solvents. Food Chemistry.

[bb0175] Szydłowska-Czerniak A., Kowaluk A., Strzelec M., Sawicki T., Tańska M. (2025). Evaluation of bioactive compounds and chemical elements in herbs: Effectiveness of choline chloride-based deep eutectic solvents in ultrasound-assisted extraction. Molecules.

[bb0180] Wang H., Chu X., Du P., He H., He F., Liu Y., Abd El-Aty A.M. (2023). Unveiling heterocyclic aromatic amines (HAAs) in thermally processed meat products: Formation, toxicity, and strategies for reduction-a comprehensive review. Food Chemistry: X.

[bb0185] Wang H., Xu Q., Zhang T., Liu J., Zeng X., Li J., Li H. (2025). Color dynamics and stabilization strategies in meat substitutes: Mechanistic insights and challenges in enhancing natural pigments. Food Bioscience.

[bb0190] Wang P., Wang K., Li T., Zhang S., Wu J., Wu F., Fang G., Wu C., Liu X. (2026). Ultrasonic-assisted deep eutectic solvents extraction of saponins from Strobilanthes sarcorrhiza: Process optimization and mechanistic insights. Chemical Engineering Journal.

[bb0195] Wang Q., Cheng W., Zhang Y., Kang Q., Gowd V., Ren Y., Cheng K.W. (2021). A novel potent inhibitor of 2-amino-1-methyl-6-phenylimidazo[4,5-b] pyridine (PhIP) formation from Chinese chive: Identification, inhibitory effect and action mechanism. Food Chemistry.

[bb0200] Xu Y., Cheng Y., Zhu Z., Guo H., Bassey A.P., Huang T., Huang M. (2023). Inhibitory effect of mulberry leaf (Morus alba L.) extract on the formation of free and bound heterocyclic amines in pan-fried muscovy duck (Cairina moschata) patties. Food Control.

[bb0205] Xu Y., Jiao Y., Luo J., He Z., Zeng M., Shen Q., Quan W. (2022). The influence of deep eutectic solvents extract from ginger on the formation of heterocyclic amines and advanced glycation end products in roast beef patties. Foods.

[bb0210] Yan Y., Zhou Y., Huang J., Wan X., Zeng M., Chen J. (2022). Influence of soybean isolate on the formation of heterocyclic aromatic amines in roasted pork and its possible mechanism. Food Chemistry.

[bb0215] Yu X., Zhao Z., Yan X., Xie J., Yu Q., Chen Y. (2023). Extraction optimization of tea saponins from Camellia oleifera seed meal with deep eutectic solvents: Composition identification and properties evaluation. Food Chemistry.

[bb0220] Yücel T., Kamiloğlu A., Kutlu N. (2025). Influence of ultrasonic-assisted deep eutectic solvent extraction on bioactive compounds from germinated mung beans (Vigna radiata) and greenness assessment. Microchemical Journal.

[bb0225] Zeng M., Li Y., He Z., Qin F., Chen J. (2016). Effect of phenolic compounds from spices consumed in China on heterocyclic amine profiles in roast beef patties by UPLC-MS/MS and multivariate analysis. Meat Science.

[bb0230] Zeng M., Wang J., Zhang M., Chen J., He Z., Qin F., Xu Z., Cao D., Chen J. (2018). Inhibitory effects of Sichuan pepper (Zanthoxylum bungeanum) and sanshoamide extract on heterocyclic amine formation in grilled ground beef patties. Food Chemistry.

[bb0235] Zhang H., Lv X., Su W., Chen B.H., Lai Y.W., Xie R., Cao H. (2024). Exploring the roles of excess amino acids, creatine, creatinine, and glucose in the formation of heterocyclic aromatic amines by UPLC-MS/MS. Food Chemistry.

[bb0240] Zhang K., Huang J., Wang D., Wan X., Wang Y. (2024). Covalent polyphenols-proteins interactions in food processing: Formation mechanisms, quantification methods, bioactive effects, and applications. Frontiers in Nutrition.

[bb0245] Zhang L., Tu Z.c., Yuan T., Wang H., Fu Z.f., Wen Q.h., Wang X.q. (2014). Solvent optimization, antioxidant activity, and chemical characterization of extracts from *Artemisia selengnesis* Turcz. Industrial Crops and Products.

[bb0250] Zhang M., Shuai X., Wei Z., Dai T., Wei C., Li Y., He J., Du L. (2024). Characterization, antioxidant and antitumor activities of phenolic compounds from *Amomum villosum* Lour. Frontiers in Nutrition.

[bb0255] Zhang S., Wang R., Chu J., Sun C., Lin S. (2023). Vegetable extracts: Effective inhibitors of heterocyclic aromatic amines and advanced glycation end products in roasted mackerel. Food Chemistry.

[bb0260] Zhou Y., Ma Y., Ma Z., Ma Q., Li Z., Wang S. (2024). Theoretical exploration of the phenolic compounds' inhibition mechanism of heterocyclic aromatic amines in roasted beef patties by density functional theory. Food Research International.

