# Supplementary material for: Amomum villosum Lour. Polyphenols extract: Effective inhibitors of heterocyclic aromatic amines in grilled beef patties
Source: Food Chem X. 2026 May 5;36:103947. doi: 10.1016/j.fochx.2026.103947 (PMC13208090; doi:10.1016/j.fochx.2026.103947)
Supplement: Supplementary file 1 — Supplementary material [file mmc1.docx]

**Supporting Information**

***Amomum villosum* Lour. polyphenols extract: Effective inhibitors of heterocyclic aromatic amines in grilled beef patties**

Yuhao Huang^a, 1^, Yanan Liu^a, c 1^, Bao-Zhu Jia^b^, Zhen-Lin Xu^a^, Lin Luo^a, *^

^a^ Guangdong Provincial Key Laboratory of Food Quality and Safety, South China Agricultural University, Guangzhou 510642, China

^b^ College of Biology and Food Engineering, Guangdong University of Education, Guangzhou 510303, China

^c^ Guangdong Research Center for Rural Policy, South China Agricultural University, Guangzhou 510642, China

^1^ These authors contributed equally to this work and should be considered co-first authors.

**Corresponding author. Lin Luo: [lin.luo@scau.edu.cn](mailto:lin.luo@scau.edu.cn)*

**Table S1**

Calibration curves of 13 HAAs standards.

| **HAA** | **Calibration curve** | **R^2^** |
| --- | --- | --- |
| 4,7,8-TriMeIQx | y = 1736.09x + 9636.46 | 0.9996 |
| 4,8-DiMeIQx | y = 8295.10x + 74942.45 | 0.9988 |
| 7,8-DiMeIQx | y = 8284.38x + 58349.12 | 0.9993 |
| 8-MeIQx | y = 2471.12x + 14170.47 | 0.9993 |
| AαC | y = 14429.35x + 89410.08 | 0.9994 |
| Glu-P-1 | y = 5821.48x + 4846.92 | 0.9999 |
| Glu-P-2 | y = 9103.47x + 2903.07 | 0.9999 |
| Harman | y = 8520.83x + 18674.95 | 0.9998 |
| IQx | y = 5979.99x + 44376.28 | 0.9991 |
| MeIQ | y = 10313.79x + 8368.48 | 0.9999 |
| Norharman | y = 5091.07x + 14591.85 | 0.9998 |
| PhIP | y = 4233.16x + 20946.56 | 0.9994 |
| Trp-P-1 | y = 12291.97x + 7094.80 | 0.9998 |
